# Copious Podocyturia without Proteinuria and with Normal Renal Function in a Young Adult with Fabry Disease

**DOI:** 10.1155/2015/257628

**Published:** 2015-05-21

**Authors:** H. Trimarchi, R. Canzonieri, A. Muryan, A. Schiel, A. Araoz, M. Forrester, A. Karl, F. Lombi, J. Andrews, V. Pomeranz, T. Rengel, E. Zotta

**Affiliations:** ^1^Nephrology Service, Hospital Británico de Buenos Aires, Perdriel 74, 1280 Buenos Aires, Argentina; ^2^Laboratory Services, Hospital Británico de Buenos Aires, Perdriel 74, 1280 Buenos Aires, Argentina; ^3^IFIBIO Houssay, UBA CONICET, Facultad de Medicina, Universidad de Buenos Aires, Paraguay 2155, 1425 Buenos Aires, Argentina

## Abstract

The time for starting a patient with Fabry disease on enzyme replacement therapy is still a matter of debate, particularly when no overt classical clinical signs or symptoms are present. With respect to Fabry nephropathy, a dual problem coexists: the reluctance of many nephrologists to start enzyme replacement infusion until signs of renal disease appear as the appearance of proteinuria or an elevation in serum creatinine and the lack of validated biomarkers of early renal damage. In this regard, proteinuria is nowadays considered as an early and appropriate marker of kidney disease and of cardiovascular morbidity and mortality. However, in this report we demonstrate that podocyturia antedates the classical appearance of proteinuria and could be considered as an even earlier biomarker of kidney damage. Podocyturia may be a novel indication for the initiation of therapy in Fabry disease.

## 1. Introduction 

Fabry disease is a chromosome X-linked hereditary disease with systemic involvement, mainly affecting the cardiovascular, renal, and neurologic systems. Despite specific replacement therapy, renal involvement is progressive [1]. The proper time for the initiation of enzyme replacement therapy in Fabry disease is still a matter of debate. Despite growing evidence that supports early pharmacologic intervention and adjusted doses to body mass index even in asymptomatic subjects [2], the majority of physicians withhold treatment until clinical evidence of organ involvement is evident. With respect to Fabry nephropathy, a dual problem coexists: the reluctance of many nephrologists to start enzyme replacement infusion until signs or symptoms of renal disease appear and the lack of biomarkers of early renal damage. In this regard, proteinuria is considered as an early and appropriate marker of kidney disease and of cardiovascular morbidity and mortality and a target to be improved. However, the appearance of proteinuria may be denoting an already damaged and denuded filtration barrier due to the reduction of podocytes, which are incapable of undergoing mitosis under normal conditions [3]. Therefore, in many situations, proteinuria would be a late biomarker of glomerular damage. In this report, we demonstrate the presence of increased podocyturia in a young adult with Fabry disease and the absence of proteinuria and stage I chronic kidney disease, suggesting that the detachment of damaged podocytes may precede proteinuria.

## 2. Case Presentation

The diagnosis of Fabry disease was made in an 18-year-old male who suffered from acroparesthesias, decreased sweating, and frequent episodes of diarrhea. The test for *α*-galactosidase disclosed decreased enzyme activity, 0.1 nmol/hour/liter (normal > 4 nmol/hour/liter). A novel mutation [c.100A>G (p.N34D)] was identified in the gene of *α*-galactosidase A, diagnosed by sequential analysis. The laboratory results were unremarkable, with a creatinine clearance of 115 mL/min, 24-hour urinary albumin excretion 19 mg/day. A renal ultrasound was normal.

**Figure 1:**
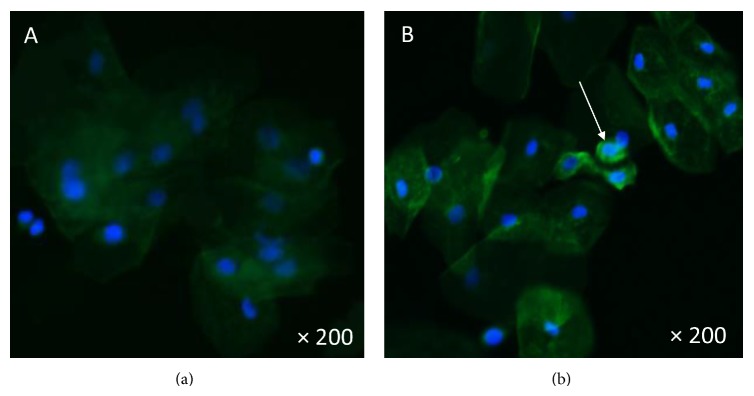
(a) Urine specimen from a control. Tubular cells are observed. No podocytes were identified. (b) The arrow indicates the presence of podocytes, as bright green fluorescent cells. Fluorescent microscopy ×200.

The podocyte count was assessed by counting in urinary smears the number of cells in 10 microscopy fields of ×20. The podocyte count was 1.6 cells per ×20 field; the number of podocytes per gram of urinary creatinine was 133, and the number of podocytes/100 mL of urine was 8. Podocytes were identified by tagging synaptopodin (ab109560 Alexa Fluor, Abcam, Cambridge, United Kingdom), a specific marker of podocytes, to establish their identity by immunofluorescence techniques using a secondary antibody (Alexa Fluor 488, Abcam, Cambridge, United Kingdom). The smears were analyzed employing an epifluorescence microscopy, Nikon Eclipse E 200. This result was compared with 5 controls (Figure 1(a), one control; Figure 1(b), Fabry patient): 3 males and 2 females, mean age: 22 ± 7.2 years with no past history of morbidities; creatinine clearance: 108 mL/min; mean 24-hour urinary albumin excretion: 88 ± 11 mg/day. The mean podocyte count was 0.12 ± 0.1 cells per ×20 field while the mean number of podocytes per gram of urinary creatinine was 10.7 and the mean number of podocytes/100 mL of urine was 1.1. The patient declined to undergo a kidney biopsy but accepted enzyme replacement therapy intravenously with agalsidase beta 1 mg/kg every fortnight (Fabrazyme, Genzyme Corp., Cambridge, MA, USA) and enalapril 5 mg/day orally.

## 3. Discussion

Fabry disease is an X-linked genetic disorder of glycosphingolipid catabolism resulting from deficient activity of the lysosomal enzyme *α*-galactosidase A. As a consequence, neutral glycosphingolipids, mainly globotriaosylceramide (GL-3), accumulate in a variety of cells and tissues, leading to a wide clinical spectrum of clinical manifestations [1, 4]. Chronic kidney disease (CKD) is a prominent feature of Fabry disease [1, 4, 5] that accounts for 0.01% of end-stage kidney disease patients enrolled in European and US dialysis registries [6, 7]. However, enzymatic screening studies suggest that the true prevalence for male dialysis patients may be 10- to 100-fold higher [8, 9]. At the cellular renal biotype level, podocytes, endotelial cells, tubular cells, and mesangial cells are injured and, consequently, the glomerular basement membrane and the interstitium are involved, resulting in proteinuria and eventually in renal failure. As we previously outlined [10, 11], the suggested mechanisms of renal injury in Fabry disease include vascular compromise secondary to deposition of GL-3 within the arterial wall, which should be considered as a* first hit*, with a concomitant decrease in endotelial nitric oxide synthesis and a tendency to microthrombotic events, podocyte injury, and detachment with secondary glomerulosclerosis, and tubular atrophy and interstitial fibrosis [12]. In Fabry disease, podocytes are special targets with important implications in prognosis. It has recently been demonstrated that in* in vitro* cultured human podocytes the accumulation of the lysosomal enzyme *α*-galactosidase A substrates such as GL3 or lyso-GL3 may lead to the secretion of transforming growth factor-*β* 1 and the triggering of profibrotic pathways [13]. Moreover, autophagy dysregulation has also been implicated in podocyte damage in Fabry disease due to the inhibition of mammalian target of rapamycin (mTOR), a key enzyme that regulates autophagy [14]. Apparently, the accumulation of GL3 is accompanied by an increase of autophagosomes and a loss of mTOR kinase activity, a negative regulator of autophagy [14]. Autophagy may therefore contribute to podocyte depletion and proteinuria. Albeit a specific treatment for the disease exists, proteinuria frequently persists, particularly as renal disease worsens [15]. 

In this respect, despite the fact that proteinuria is a useful marker of kidney disease and of glomerular injury, it is not specific of the stage of kidney damage, as it can be found at any stage of CKD. In our patient, proteinuria was negative and kidney function was normal. However, an excessive loss of podocytes in the urine could have been indicating an established structural glomerular abnormality and would herald the ulterior appearance of proteinuria. In addition, as podocytes do not replicate, once podocytes are detached from the glomerular basement membrane, the filtration barrier becomes denuded and proteinuria ensues when contiguous podocytes are unable to cover the function of the lost ones. It has been reported that when the population of podocytes per glomerulus is reduced to a 20–40%, the process of glomerular obliteration is initiated [3, 16]. Finally, it has been calculated that around 400 podocytes are lost in the urine every day, which explains the podocyturia the control patient also presents, but of lower quantity [3, 16].

It is also critical in glomerular diseases, and particularly in Fabry disease for which a specific therapy is available, to start treatment as early as the diagnosis is made, in order to avoid GL-3 accumulation and cellular damage [2]. Tøndel et al. have separately demonstrated that, in Fabry patients, early and then continuous enzyme replacement therapy leads to a gradual clearance of zebra bodies in endotelial and tubular cells and a partial improvement in GL-3 accumulation in podocytes, which would then translate into a decreasing amount of proteinuria and a better kidney function survival [2, 17]. In summary, enzyme replacement intervention should be started as the diagnosis is made, because proteinuria is a delayed marker of advanced glomerular damage in Fabry disease, as our case report and other works demonstrate [2, 12].

Why do podocytes detach in Fabry disease? The *α*v*β*3 integrin (also known as the vitronectin receptor) anchors the podocyte to the glomerular basement membrane; when activated, it causes podocyte contraction and eventually contributes to the detachment of the cell from the glomerulus and its appearance in the urine [17]. Interestingly enough, Utsumi et al. have reported that the urinary excretion of *α*v*β*3 integrin is elevated in subjects with Fabry disease. Increased expression of the *β*3 component was observed in glomerular epithelial cells and in Bowman's capsular epithelial layer and tubular cells, and the amount of vitronectin (a molecule involved in adhesion and fibrinolysis) was moderately increased in the kidney from Fabry patients. The urinary excretion of the integrin *α*v*β*3 was also increased and its expression was also observed in Fabry kidney tissues, apparently due to the accumulation of GL3. Therefore, the expression of the integrin *α*v*β*3 may be involved in podocyte contraction and eventual detachment from the glomerular basement membrane and could be another pathophysiological cause of proteinuria, finally contributing to the progression of renal injury in Fabry disease [18].

Despite the fact that treatment with agalsidase and angiotensin converting enzyme inhibitors is begun, proteinuria may persist in Fabry disease, particularly as renal disease worsens [11, 15]. This could be due to the podocytopenia that had not been noticed since early stages of the disease. Therefore, we suggest that podocyturia could be employed to assess the level of kidney damage despite the absence of proteinuria and to hasten the initiation of agalsidase therapy, which would be beneficial for the patient [2]. The technique is simple but time-consuming; it needs to be validated, and the costs may be lower as the test is spread out. In our patient, the podocyte loss was lower than that reported in normal subjects [3]. However, it was considerably higher than that found in our controls. This may be due to several facts, including the lack of validation studies, the marker employed to identify podocytes, the observer expertise, and the technique employed. All these considerations may explain the discrepancy encountered in this report, remarking the necessity of an assay standardization of podocyturia. Moreover, the search for podocytes in the urine is a noninvasive tool that could serve as a complement to a kidney biopsy, as a marker of response to therapy, or even to reinforce the necessity of a kidney biopsy when a formal patient reluctance for it exists.
